# Translating Theory into Practice: Lessons Learned from Developing a Program Model to Foster Resiliency in Expectant and Parenting Youth

**DOI:** 10.1007/s10995-020-02890-x

**Published:** 2020-02-17

**Authors:** Lissa Pressfield, Mary Campa, Karen Ramstrom, Sangi Kabadi, Catherine Lopez

**Affiliations:** 1 California Department of Public Health, Maternal, Child and Adolescent Health Division, PO Box 997377, MS 0510, Sacramento, CA 95899 USA; 2 Shasta County Health and Human Services Agency, 2650 Breslaur Way, Redding, CA 96001 USA; 3 California Department of Public Health, Women, Infants and Children (WIC) Division, 3901 Lennane Drive, Sacramento, CA 95834 USA

**Keywords:** Program development, Positive youth development, Resiliency, Expectant and parenting youth, Evidence informed

## Abstract

**Purpose:**

This paper describes the approach used to develop the Adolescent Family Life Program (AFLP) Positive Youth Development (PYD) Model within the structure of an existing state government-run program.

**Description:**

The California Department of Public Health, Maternal, Child and Adolescent Health (CDPH/MCAH) Division undertook an innovative approach to develop a program model to help expectant and parenting youth build resilience. CDPH/MCAH started by assessing existing program efforts and theory to develop and test new strategies in the field, structure a program model, and build toward broader expansion and sustainability. CDPH/MCAH engaged local organizations from across the state, their staff and enrolled youth, experts, and evaluators in an iterative program development process to standardize an effective model that could be replicated and evaluated.

**Assessment:**

Key lessons for program developers and administrators are to ensure adequate staffing with diverse expertise related to the topic and content to support the multiple components of program development and implementation, evaluation, and training; identify the guiding theory and framework early and link them with clearly articulated core components to ensure the final model reflects the intended purpose and is structured to support implementation; engage implementation staff on the ground and focus early and often on processes for supporting people through change.

**Conclusion:**

The lessons learned can guide others working with existing programs to develop standardized program models or translate new science and theory into practice.

## Significance

Although state agencies often fund and oversee evidence-based public health program models, expanding to program model development presents unique opportunities for broad impact. The California Department of Public Health undertook a process to develop a program model for expectant and parenting youth within an existing program. The process involved extensive engagement of local implementing agencies, youth participants, and experts. The lessons learned can guide others in considering program improvements based on new science and compelling theory. They also reveal the value of a participatory process, which requires intention around managing change and developing systems for supporting implementation and evaluation.

## Purpose

Expectant and parenting youth (EPY) often face unique social, economic, and health challenges that can limit their opportunities for success. These challenges may include factors such as inadequate or unsafe living environments, racial and income inequalities, and insufficient access to health care and education (Pinzon and Jones 2012; Leplatte et al. 2012). Compared to births to adult women, infants born to adolescents are at greater risk for preterm birth, low birthweight, and death during infancy (Santelli et al. 2017; Ventura et al. 2011). Although early childbearing is associated with negative life course outcomes, research suggests that many of the negative effects are not caused by early childbearing but rather exacerbated by risk factors, such as those described above, that existed prior to pregnancy (Hotz et al. 2005; Kearny and Levine 2012; Kane et al. 2013; Santelli et al. 2017). Early childbearing can lead to cycles of poverty and disadvantage—yet with effective support, trajectories and outcomes can improve. Research shows that becoming a parent at a young age can act as a motivating factor for young people to pursue opportunities to better care for their children (Spear and Lock 2003). Programs tailored to meet EPY’s needs and goals can help to address the effects of preexisting risk factors and challenges of early parenthood to improve life course outcomes for young families.

Recognizing the unique and critical needs of EPY and the lack of evaluated programs designed to support them (Chrisler and Moore 2012), the California Department of Public Health, Maternal, Child and Adolescent Health (CDPH/MCAH) Division launched an effort to design an evidence-informed program model coupled with rigorous evaluation as a strategy to expand effective approaches and meet the needs of EPY. The effort involved engaging staff and youth participants from 11 state-funded Adolescent Family Life Program (AFLP) local agencies, subject-matter experts, and evaluators in an iterative program development process. The intent of the process was to learn about best practices, identify challenges, develop and pilot new strategies, and build an effective model for EPY. A unique feature of this project is the scale and scope of the effort and potential impact. California is largest state by population size, with growing diversity and more than 19,000 births annually to adolescents 19 years old and younger (CDPH 2017). CDPH/MCAH worked within the existing state AFLP infrastructure to develop a standardized approach to promote resilience in EPY that could be integrated into the broader program infrastructure for expansion and evaluation. This paper describes the process used and lessons learned by CDPH/MCAH in developing the new model, called the AFLP Positive Youth Development (PYD) Model, while sustaining services for youth in areas of high need throughout the state.

## Description

Over the past decade, public health has seen increased focus on evidence-based program models (EBPMs) for improving health and well-being. EBPMs are models found to be effective at improving specific outcomes based on rigorous research (Cooney et al. 2007). Often, funding to implement EBPMs is better justified because replication with fidelity can increase the likelihood of achieving the same health outcomes as the original research. Because of the value of evidence-based programming, in the mid-2000s, CDPH/MCAH assessed research on programs for expectant and parenting youth. CDPH/MCAH found few EBPMs for EPY and none created from a PYD framework, a set of concepts research shows positively impacts youth (Lerner and Lerner 2009). PYD approaches are strengths based and goal oriented, value youth voice and engagement, focus on empowerment and opportunity, ensure developmental and cultural responsiveness, and rely on the protective nature of caring youth-adult relationships and supportive networks to promote positive health, educational, and social outcomes.

CDPH/MCAH also assessed its own longstanding case management program for EPY, the Adolescent Family Life Program (AFLP). In 2008–2009, AFLP suffered major budget cuts triggered by the economic downturn. To sustain funding and build public confidence for the program, CDPH/MCAH needed to show that program activities resulted in positive outcomes. However, it was difficult to identify successful strategies, measure their effectiveness, and ultimately demonstrate program impact because of the substantial variation in how AFLP was implemented in agencies across the state. For example, although all agencies worked with youth to develop individual service plans, some agencies implemented a crisis management model, whereas others focused more broadly on goals and youth development. Additionally, although the program required monthly visits with youth, the number of visits each youth received every month varied by agency. A standardized program model and data collection processes were needed.

In 2010, CDPH/MCAH received an Office of Population Affairs (formerly the Office of Adolescent Health) Pregnancy Assistance Fund (PAF) grant to develop and implement a new program model for EPY in California. The PAF project provided CDPH/MCAH with resources to build a team to develop and pilot a model leveraging the existing AFLP and a formative evaluation process. CDPH/MCAH’s goal was to increase and sustain support for California’s youngest families through the development of a standardized model that could reliably produce meaningful results.

This section describes the 5-year, nonlinear process of securing resources, building a team, and developing the AFLP PYD Model across three project phases: (1) early program development, (2) iterative pilot implementation and formative evaluation, and (3) formalizing the model structure for expanded implementation and evaluation. (See Table 1 for a summary of the opportunity and program development phases.) The authors present the process and lessons learned from their experience developing the program and provide a potential road map and ideas for others engaging in similar processes.

**Table 1:** Overview of the Adolescent Family Life Program (AFLP) Positive Youth Development (PYD) Model development activities and lessons learned

Opportunity and program development components	Key points and activities	Links to the lessons learned
Opportunity: Pregnancy Assistance Fund Grant	• CDPH/MCAH was awarded a PAF grant, which provided an opportunity to develop, implement and evaluate a program model specifically for expectant and parenting youth • In 2010–2011, CDPH/MCAH hired two full time equivalent program staff to lead program development and contracted with 11 local agencies and a University of California, San Francisco research team to support program development and external formative evaluation • In 2013, MCAH added an additional .75 FTE state staff to the project with expertise in developing, monitoring, and evaluating evidence-informed program models • The pre-existing AFLP infrastructure and the state leadership were key to the project	Lesson 1: *Ensure Adequate Staffing to Support the Multiple Components of Program Development, Implementation and Monitoring* When developing a new program model, consider the diverse expertise needed from the beginning. At a minimum, it is essential to have people with the following expertise: topical, program development and implementation, monitoring and evaluation, and training. This can either be through governmental staff or contractors. Careful thought should be given to all components of program development and mechanisms should be in place to hire internal staff or contract staff, based on the needs and goals of the project. Given the relatively lengthy process of program development, efforts to hire staff with a sustained commitment to the project can help to ensure continuity in development.
Early program development (2010–2012)	The CDPH/MCAH project team: • Developed the vision and program strategies (case management, positive youth development, motivational interviewing, and life planning) • Conducted literature reviews on positive youth development, reproductive life planning, goal setting, motivational interviewing, and evidence-based program models for EPY and other youth populations • Selected 11 of the 34 AFLP sites to participate in the program development process as pilot sites • Learned about best practices among pilot sites through environmental scans and site profiles • Provided foundational training	Lesson 2: *Identify the Guiding Theory and Framework Early and Link them with Clearly Articulated Core Components* Identifying the theory and framework early provides the foundation for developing all program model elements and supports the process of identifying areas where training and technical support are needed. Having a strong foundation to link all parts of program development helps support and justify the many changes that arise from a participatory and iterative development process. Articulating clear and specific core components leads to a program structure that can be consistently implemented and evaluated.
Iterative Pilot Implementation and Formative Evaluation (2011–2013)	The CDPH/MCAH project team: • Developed/adapted tools and processes for a standardized program model • Selected a foundational PYD framework: *Resiliency in Action* (Bernard 2004) • Worked to take the theory and operationalize it in case management practice • Produced guides and trainings for implementers to help with the paradigm shift • Led an iterative development process with the pilot sites • Sent tools/processes out into the field (with training/technical assistance/written guidance) to be implemented with youth in AFLP • Gathered feedback (through interviews, surveys, site visits, focus groups, group calls, etc.) and produced recommendations • Made revisions (further engaging local agencies through work groups, site visits and calls, when needed) and then sent revised materials out into the field for further piloting. The process was repeated through multiple cycles	Lesson 3: *Engage Implementing Staff on the Ground and Focus Early, and Often, on Processes for Supporting People through Change* The AFLP PYD Model program development occurred through pilot implementation in an existing program. The model was a paradigm shift for some agencies and the iterative feedback/implementation cycle led to revisions and frequent changes for implementers. Lesson 3 speaks to the importance of intentionally supporting the change process as the new program model elements and supporting systems are developed and refined.
Formalizing Model Structure for Expanded Implementation and Evaluation (2013–2014)	CDPH/MCAH further synthesized formative evaluation results and produced an evaluation-ready version of the AFLP PYD Model. This included: • A formalized model structure, program tools and materials, guiding documents, trainings, fidelity and outcome monitoring system, and a quality improvement process • CDPH/MCAH launched AFLP PYD implementation with 14 additional AFLP sites • December 2014, Mathematica launched the Positive Adolescent Futures study. Results are anticipated in 2020	Lesson 1: *Ensure Adequate Staffing to Support the Multiple Components of Program Development and Implementation* When developing a new program model diverse expertise is needed from the beginning to help ensure that the final product can be implemented and evaluated across settings. Lesson 2: *Identify the Guiding Theory and Framework Early and Link them with Clearly Articulated Core Components* The guiding theory and framework are foundational for the development work. Specific core components that articulate the content, logistics and pedagogy are necessary to support implementation that can be monitored and evaluated across multiple agencies/settings.

From 2010 to 2012, during the early program development phase, CDPH/MCAH developed the vision for building the program model by exploring existing local practices in AFLP and reviewing literature on effective strategies to support youth and new parents. Four strategies emerged and guided development of the AFLP PYD Model: (1) case management, (2) positive youth development approaches, (3) motivational interviewing (MI), and (4) reproductive life planning. Case management is a best practice for supporting EPY in meeting their unique needs and goals (U.S. Department of Health & Human Services, Family and Youth Services Bureau 2012). Substantial research shows that PYD approaches are beneficial for improving health, social, and education outcomes among youth (Gloppen et al. 2010; Lerner and Lerner 2009; Markham et al. 2010). Motivational interviewing, a form of collaborative conversation designed to strengthen one’s commitment to behavior change, also showed effectiveness with adolescents (Gold and Kokotailo 2007; Lundahl and Burke 2009; Naar-King and Suarez 2011; Rotz et al. 2016). MI was particularly relevant because many AFLP case managers were already trained and using the techniques. The final strategy, reproductive life planning, eventually broadened to overall life planning, incorporated a process to help individuals create a plan with goals based on their values, strengths, and resources in the context of their life circumstances (Johnson et al. 2006).

During this first phase, CDPH/MCAH selected 11 of the 34 local AFLP agencies to work with to develop a new model (referred to as pilot sites). CDPH/MCAH worked with the pilot sites on environmental scans and site profiles to learn about best practices and existing efforts implemented in the field. CDPH/MCAH also trained the pilot sites on the foundational strategies for the model development—PYD, motivational interviewing, and life planning.

From 2011 to 2013, during the second project phase—iterative pilot implementation and formative evaluation—CDPH/MCAH and the pilot sites engaged in a cyclical program development process to structure and operationalize strategies and best practices identified as effective with youth. This process included (1) developing or adapting program model elements with local staff, youth, and expert consultants; (2) sending the model elements into the field for local agencies to implement with youth in their existing AFLP; (3) gathering feedback from local agencies (staff and youth) about the implementation experience through formative evaluation; (4) adapting or repackaging the model elements; and then (5) sending the revisions back from the state to local agencies for implementation. This cycle was repeated multiple times. After each major revision, CDPH/MCAH provided case managers and supervisors with training, guidance for implementation, and revised data collection processes that aligned with the changes in implementation.

The first tool pilot tested was a reproductive life planning tool called the *My Life Plan,* originally developed by one of the AFLP sites. Case managers quickly focused on implementing and providing feedback on the *My Life Plan* and expressed challenges with integrating PYD concepts into their daily work. CDPH/MCAH received feedback about the need to clarify concepts of PYD and resiliency, particularly in the context of helping youth to meet their basic needs and work toward personal goals. Integrating PYD in the context of every interaction required a shift in the program paradigm from a traditional framework—where professionals intervene to fix specific issues—to one based on PYD principles, which emphasizes young people’s abilities and supports them in building strengths, skills, and resilience.

In response to the challenges faced by the 11 pilot sites, CDPH/MCAH recognized the need for a clearer theoretical framework to guide all elements of program development. CDPH/MCAH worked with youth development experts and identified Bonnie Bernard’s *Resiliency in Action* (Bernard 2004) framework as the best fit to meet the goals of AFLP. Bernard’s framework holds that by building protective factors, youth can meet their basic needs and build resilience, which results in improved social, academic, and health outcomes (Bernard 2004). Having in mind the unique challenges expressed by local case managers and agencies, CDPH/MCAH developed a program guide that outlined how case managers working with expecting and parenting youth could apply the concepts from Bernard’s resiliency framework. CDPH/MCAH also released revised program content to support the integration of PYD concepts including strengths, values, and goal setting.

A key feature of the program development process was collecting feedback from the local agencies as program materials were developed and revised. CDPH/MCAH maintained regular communication with the pilot sites through monthly group calls where agencies implementing AFLP PYD could come together for peer support, monthly one-on-one technical assistance calls, and other trainings and work groups. CDPH/MCAH also worked with an external evaluation partner, the University of California, San Francisco (UCSF), to collect information about implementation through semi-structured telephone and in-person interviews and online surveys with local case managers and supervisors, observations of youth-case manager visits, focus groups with youth, and training evaluations. The evaluation resulted in anonymous feedback addressing the utility of the newly developed program materials; factors that impeded or facilitated implementation; and summaries of local staffs’ understanding of the underlying strategies (e.g., positive youth development, motivational interviewing, and life planning). At the end of each cycle of formative feedback, CDPH/MCAH received an evaluation report with key findings and recommendations for program improvements. The formative evaluation (phase 2) provided CDPH/MCAH with the information needed to make revisions and ultimately to formalize the content, structure, and packaging of the AFLP PYD Model for expanded implementation and evaluation (phase 3). All evaluation work related to the project described in this article was approved by the Institutional Review Board of the State of California and was completed separately and prior to the federal evaluation, the Positive Adolescent Futures study, documented by Asheer and colleagues in this journal supplement (Asheer et al. in press), with additional findings anticipated in 2020.

In 2013, as the formative evaluation was concluding, CDPH/MCAH secured a second PAF grant. Although key parts of the model were developed—including the foundational resiliency framework, program phases, and key activities/tools—the model was not yet packaged to support standardized implementation, expansion, and evaluation. During an extension year from the first PAF grant (2013–2014), which overlapped with the second grant, CDPH/MCAH was selected for the federal evaluation of the AFLP PYD Model conducted by Mathematica (Asheer et al. in press). The federal evaluation provided the impetus to add state staff to the project who had expertise in developing, monitoring, and evaluating evidence-informed program models and expedited the structuring and packaging of the full AFLP PYD Model. As part of the model packaging, CDPH/MCAH articulated core components of the model in terms of logistics (required structural features, such as setting and number of visits); pedagogy (required implementation strategies, such as motivational interviewing); and content (required activities and conversations, such as those around health and education). The core components resulted from what research indicated should drive change and what the pilot testing and formative evaluation revealed was relevant and feasible in the field. The AFLP PYD Model was structured into 24 face-to-face visits between the case manager and the youth within a 12-month period across four distinct program phases. CDPH/MCAH created a visit-by-visit guide for local agencies that articulated the specific core components and the purpose of the activities related to the resiliency framework and program goals. The visit guide also provided case managers with parameters about where and how to be flexible and responsive to the needs of youth, in relation to model guidelines, while maintaining fidelity to the model. From December 2014 through June 2015, CDPH/MCAH expanded the AFLP PYD Model implementation to an additional 14 local sites, many of which participated in the federal evaluation conducted by Mathematica.

## Assessment

CDPH/MCAH offers the following key lessons that emerged through the experience of developing the AFLP PYD Model. The lessons are particularly relevant when developing a program model with extensive engagement from program implementers and participants. In addition, developing and implementing a new model in the context of an existing program presents unique challenges and requires intentional strategies to support success. CDPH/MCAH offers three key lessons that emerged throughout the program development process for consideration by others (see Table 1 for a summary of the links between program development components and the lessons).

### Lesson One: Ensure Adequate Staffing to Support the Multiple Components of Program Development, Implementation, and Monitoring

The need to ensure adequate staff capacity (number and specific capabilities) to support program development along with routine program monitoring and technical support should not be underestimated. For this type of program development, it is essential to have staff in place that have topical expertise, knowledge of program development and implementation of evidence-informed/based models, monitoring and evaluation expertise, and the ability to train and write clear guidance for implementation. Staff with these areas of expertise should be engaged from the beginning of the project, when possible. From a state perspective, it is also important to have efficient contracting mechanisms and supportive funders that provide the opportunity to engage people with the needed expertise (e.g., to fund local implementers and content experts, evaluators, and trainers, if the expertise is not available at the state).

Early resourcing of the AFLP PYD Model development project focused heavily on conceptual strategies and program material development, including contracted research staff to engage in formative evaluation with local agencies and conduct literature reviews. The state staff translated the research and findings from the field into a packaged model that could be replicated and developed tools to monitor and evaluate implementation. CDPH/MCAH started this work with a small team that gradually grew to include additional expertise. Although the state team was able to respond to formative feedback, revise materials, develop and conduct trainings, update data systems, and provide tailored support to each implementation site, there were substantial challenges and setbacks along the way that may have been alleviated or lessened by having the diverse expertise needed at the project’s outset. Other challenges included staff turnover and extended leaves, which reduced the continuity of the development process and resulted in the loss of particular expertise. These contributing factors are often impossible to control but affect program development. Sustained commitment from CDPH/MCAH leadership and the federal funder, coupled with gradually growing the state team’s expertise and the ongoing dedication from local agencies and UCSF, supported continued success of the development process.

### Lesson Two: Identify the Guiding Theory and Framework Early and Link Them with Clearly Articulated Core Components

Without a clear scientific framework guiding program development, the iterative nature of the process can dilute the final product. Program development research suggests that a set of core components is essential (Blase and Fixsen 2013). CDPH/MCAH’s experience reaffirms this finding. In this case, the defined strategies (PYD, MI, and life planning) and resiliency framework provided the underpinnings for the vision and practice ideas within case management. The work then focused on further operationalizing the strategies and theory by learning from the experiences in the field and defining core logistical, pedagogical, and content components. Tools and processes developed and changed over time, but with a strong foundation, vision, and leadership, CDPH/MCAH was able to build out the core components with enough specificity so the program could be implemented and evaluated across multiple program sites. The guiding documents, trainings, monitoring systems, ongoing technical assistance, and quality improvement processes supported the capacity of local staff to implement the program with fidelity.

### Lesson Three: Engage Implementing Staff on the Ground and Focus Early, and Often, on Processes for Supporting People Through Change

CDPH/MCAH developed the AFLP PYD Model through extensive engagement with local sites already implementing AFLP case management for EPY. A primary benefit to this approach was the rapid implementation and testing of model strategies and program materials by local staff and youth. Their experience, ideas, and feedback informed changes that ultimately helped ensure program activities were feasible to implement and resonated with youth. Simultaneously continuing services while piloting new and changing strategies gave rise to other challenges. In particular, local agencies expressed considerable anxiety and practical challenges related to the frequency that program tools, processes, and data collection forms changed throughout the cycles of formative feedback and model refinements. CDPH/MCAH engaged in multiple strategies to balance the needs for stability and local planning with the commitment to integrating science and local input for program improvement. Identifying local champions that demonstrated enthusiasm and proficiency helped sustain momentum in the face of challenges. CDPH/MCAH gave local champions opportunities to share experiences and strategies with others slower to adapt to changes. Acknowledging local expertise and providing the opportunity for local staff to express and discuss concerns and to link back to the program development purpose—to create and sustain an effective program for EPY—helped some people adapt. Regular technical assistance, training, site visits, guidance, resources, and ongoing communication also helped alleviate the stress and uncertainty that came with implementing a developing program model.

## Conclusion

The public expects that public health agencies will not only monitor population health but will also protect and respond, on the public’s behalf, in a responsible and transparent manner. Evidence-informed and evidence-based models are needed to improve the health and well-being of general and targeted populations. Dedicated resources are essential for supporting the related development, evaluation, and program implementation. Through the PAF project, CDPH/MCAH embarked on a process to engage and listen to the voices of local professionals and youth to develop a program that honors and builds the strengths and resiliency within young people.

The AFLP PYD Model started as an idea to integrate the latest scientific evidence for supporting EPY with promising practices implemented by local AFLP sites throughout the state. Through diverse expertise from local and state implementers, program developers, evaluators, and trainers, CDPH/MCAH operationalized, tested, and refined the concepts of PYD for EPY. The result was a structured program model with defined core components that could be implemented and evaluated across multiple settings. This project highlights the value of a participatory process as well as the challenges of simultaneously providing services while pilot testing and adapting to programmatic changes. CDPH/MCAH employed multiple strategies for supporting field staff with changing implementation. Key strategies that helped sustain commitment at the state and local levels included identification of local champions to support others in the field, frequent opportunities for support and shared learning between local implementers and state program development staff, and the iterative development of tools and systems to support implementation.

Fundamentally, program development requires developers and staff to be responsive, open minded, and adaptive—thus, nimble yet also steadfast around what is core to the work. State public health agencies are in a unique position to leverage the experience and commitment of state and local staff towards innovative solutions that can have widescale impact. The work described in this paper provides an example for other public health and social service entities that are considering taking a leadership role in designing programs or program improvements based on new science and compelling theory.
